# A *C. elegans* model for neurodegeneration in Cockayne syndrome

**DOI:** 10.1093/nar/gkaa795

**Published:** 2020-10-06

**Authors:** Amanda F C Lopes, Katarzyna Bozek, Marija Herholz, Aleksandra Trifunovic, Matthias Rieckher, Björn Schumacher

**Affiliations:** Institute for Genome Stability in Aging and Disease, Medical Faculty, University of Cologne, Joseph-Stelzmann-Str. 26, 50931 Cologne, Germany; Cologne Excellence Cluster for Cellular Stress Responses in Aging-Associated Diseases (CECAD), University of Cologne, Joseph-Stelzmann-Str. 26, 50931 Cologne, Germany; Center for Molecular Medicine (CMMC), Faculty of Medicine and University Hospital Cologne, University of Cologne, Robert-Koch-Str. 21, 50931 Cologne, Germany; Cologne Excellence Cluster for Cellular Stress Responses in Aging-Associated Diseases (CECAD), University of Cologne, Joseph-Stelzmann-Str. 26, 50931 Cologne, Germany; Institute for Mitochondrial Diseases and Aging, Medical Faculty, University of Cologne, D-50931 Cologne, Germany; Cologne Excellence Cluster for Cellular Stress Responses in Aging-Associated Diseases (CECAD), University of Cologne, Joseph-Stelzmann-Str. 26, 50931 Cologne, Germany; Center for Molecular Medicine (CMMC), Faculty of Medicine and University Hospital Cologne, University of Cologne, Robert-Koch-Str. 21, 50931 Cologne, Germany; Institute for Mitochondrial Diseases and Aging, Medical Faculty, University of Cologne, D-50931 Cologne, Germany; Institute for Genome Stability in Aging and Disease, Medical Faculty, University of Cologne, Joseph-Stelzmann-Str. 26, 50931 Cologne, Germany; Cologne Excellence Cluster for Cellular Stress Responses in Aging-Associated Diseases (CECAD), University of Cologne, Joseph-Stelzmann-Str. 26, 50931 Cologne, Germany; Institute for Genome Stability in Aging and Disease, Medical Faculty, University of Cologne, Joseph-Stelzmann-Str. 26, 50931 Cologne, Germany; Cologne Excellence Cluster for Cellular Stress Responses in Aging-Associated Diseases (CECAD), University of Cologne, Joseph-Stelzmann-Str. 26, 50931 Cologne, Germany; Center for Molecular Medicine (CMMC), Faculty of Medicine and University Hospital Cologne, University of Cologne, Robert-Koch-Str. 21, 50931 Cologne, Germany

## Abstract

Cockayne syndrome (CS) is a congenital syndrome characterized by growth and mental retardation, and premature ageing. The complexity of CS and mammalian models warrants simpler metazoan models that display CS-like phenotypes that could be studied in the context of a live organism. Here, we provide a characterization of neuronal and mitochondrial aberrations caused by a mutation in the *csb-1* gene in *Caenorhabditis elegans*. We report a progressive neurodegeneration in adult animals that is enhanced upon UV-induced DNA damage. The *csb-1* mutants show dysfunctional hyperfused mitochondria that degrade upon DNA damage, resulting in diminished respiratory activity. Our data support the role of endogenous DNA damage as a driving factor of CS-related neuropathology and underline the role of mitochondrial dysfunction in the disease.

## INTRODUCTION

Cockayne syndrome (CS) is an autosomal recessive genetic disorder with an occurrence of 2.5 cases per million births worldwide, and it is caused by mutations in the two genes *ERCC8*, also known as CSA, and *ERCC6*, commonly described as CSB, accounting for 20% and 80% of CS cases, respectively (1–3). The CSA and CSB proteins initiate transcription-coupled nucleotide excision repair (TC-NER) upon RNA polymerase II stalling to remove helix-distorting lesions such as UV-induced cyclobutane pyrimidine dimers (CPDs) (4). The spectrum of clinical features observed in CS patients ranges from cutaneous photosensitivity, retarded development, loss of subcutaneous fat, hearing and vision loss, cachectic dwarfism, stooped posture, and progressive neurodegeneration (1,3,4). Many traits of CS patients are reminiscent of age-related pathologies, classifying it as a progeroid syndrome (2).

Although the role of CSA and CSB in NER has been intensely investigated, the pleiotropic phenotypes associated with their dysfunction and the cellular and molecular defects underlying the symptoms are still poorly understood. A limited number of patients, most of which are compound heterozygotes, and the complexity and diversity of the symptoms challenge the study of the disease. It is unclear whether a single cellular process or whether a combination of events caused by various mechanisms could be contributing to the pathology differentially, synergistically and in cell type-specific ways (5). Thus, deciphering CS on a molecular level will provide further understanding of its underlying pathologies and the mechanisms of normal ageing, ultimately allowing for the design of possible intervention strategies.

Current systems for studying CS include mouse models of CSA, which recapitulate the human clinical features of UV-sensitivity (4); and the mouse model for CSB, which presents loss of spiral gangliocytes in the inner ear and cachectic dwarfism, apart from UV-sensitivity (6). *Csa*- and *Csb*-deficient mice, nevertheless, show pronounced susceptibility to skin cancer, which is not found in human cases of CS, and fail to exhibit any gross anatomical abnormalities (1,2,4,7). Neurodegenerative features commonly seen in CS patients only become apparent in mouse models with CS when these are crossed with mice with other deficiencies in the NER, such as *Xpc^−^^/^^−^* or *Xpa^−^^/^^−^* (1,8). While these mouse studies have been important for understanding some of the clinical features observed in humans, the diversity of the phenotypes observed in mammals, particularly in CS, renders the need for establishing a simple whole-organism model system. Therefore, we evaluated the CS-like model character of *Caenorhabditis elegans*, an organism that, although rudimentarily organized, contains many of the distinct cell types conducting distinct functions, as is seen in mammals, such as muscle cells, neurons, and intestinal cells (9).

*Caenorhabditis elegans* has become widely used as a simple model organism to untangle complex mechanisms underlying the DNA damage response and human diseases, particularly in the context of ageing (10). The proteins CSA and CSB are well conserved throughout evolution, and mutations in the respective genes have also been described in *C. elegans* (11,12). Nematodes that carry mutations in *csa-1* or *csb-1*, display developmental growth retardation and lifespan shortening upon UV treatment (11,13–15). Given the phenotypic parallels between the UV-induced phenotypes in the nematode and the progeroid pathologies in CS patients, we sought to establish whether the *C. elegans* model might recapitulate neurodegenerative phenotypes. Indeed, with only 302 neurons, a fully mapped connectome and availability of advanced neuroimaging methods, the nematode has proven exemplary in pursuing the mechanistic link between neural circuits and behaviour, such as locomotion (16), chemotaxis (17) and mechanosensation (18), as well as the intricate mechanisms of neurodegeneration in ageing and disease (19).

Here, we demonstrate that *csb-1* mutants exhibit progressive functional loss of sensory neuronal function that is exacerbated by UV irradiation or infliction of transcription-blocking DNA lesions by the fungal toxin Illudin M, emphasizing the causal role of DNA lesions in the pathology. We determine different types of progressive neuronal defects, with an accentuated level of beading (described as focal enlargements along the axonal process) in *csb-1* mutant animals, a phenotype, which precedes neuronal degeneration (20). Consistent with a role of mitochondrial dysfunction in CS-related functional deterioration, we observe higher levels of mitochondrial mass and a disturbed mitochondrial network. Despite those alterations, *csb-1* mutants maintain respiratory activity, while exogenous DNA damage triggers fragmentation of the mitochondrial networks and loss of respiratory activity. In summary, we propose the *C. elegans csb-1* mutant as a model to study mechanisms of CS, in particular, neuronal abnormalities.

## MATERIALS AND METHODS

### *C. elegans* strains and maintenance

*Caenorhabditis elegans* were grown at 20°C on NGM plates with *E. coli* strain OP50 (21) and grown in light protected incubators to minimize DNA lesions resulting from visible light (22). Strains used were (see Supplementary Table S1): N2 (Bristol, Wild type); RB1801, *csb-1(ok2335)* X.; CB4037, *glp-1(e2141)* III., BJS259, *glp-1(e2141)*;*csb-1(ok2335)*; to image and measure mitochondrial mass we used SJ4103, *zcls14*[myo-3::GFP(mit)] and SJ4143, *zcls17*[ges-1::GFP(mit)], and created BJS348, *zcls14[myo-3::GFP(mit)];csb-1(ok2335)*, BJS332, *zcls17[ges-1::GFP(mit)];csb-1(ok2335)* double-mutants. To image neurodegeneration we used CZ10175, *zdls5*[mec-4::GFP+lin-15(+)] I, and created BJS333, *zdls5[mec-4::GFP+lin-15(+)]I;csb-1(ok2335)*. The CSB-1::GFP rescue line was created via co-microinjection (23) of Fosmid CBGtg9050A06211D of the TransgeneOme project (24) and a pharyngeal tdTomato co-injection marker (p*_myo-2_*tdTomato into *csb-1(ok2335)*, followed by gamma radiation-induced integration of the extrachromosmal array. The resulting strain BJS960, *sbjIs59*[pBS28(p*_csb-1_*CSB-1::GFP)+pBS174(myo-2::tdTomato)] was backcrossed with the *csb-1(ok2335)* mutant three times.

### Locomotion assay

Animals were synchronized via sodium hypochlorite treatment and grown into Day 1 of adulthood. About 200 worms per strain and condition were transferred to fresh OP50-seeded and mock-treated, or treated with UVB (80 mJ/cm^2^), respectively. Videos (∼1.5 min) of worm locomotion were captured 24 h post-UVB irradiation with a Zeiss Axio Zoom V.16 microscope equipped with an Axiocam 503 mono. To quantify posture and mobility aspects of the animals we used TierpsyTracker (25). Using the software interface we adapted segmentation parameters (range of size of foreground patches, foreground-background threshold), keeping all remaining parameters at their default values. We batch-processed all video files obtaining trajectory information of the segmented worms excluding overlapping events and young individuals. We additionally removed all trajectories shorter than 5 s from further analysis. We obtained 50–123 worm trajectories per condition, with median trajectory length of 24 s. We next extracted posture and motility features of the tracked worms quantified by the TierpsyTracker software, such as speed, angular velocity, path range, etc. and compared their distribution between wild type, *csb-1* mutant and the CSB-1::GFP rescue line.

### Chemotaxis assay

**Figure 1.**
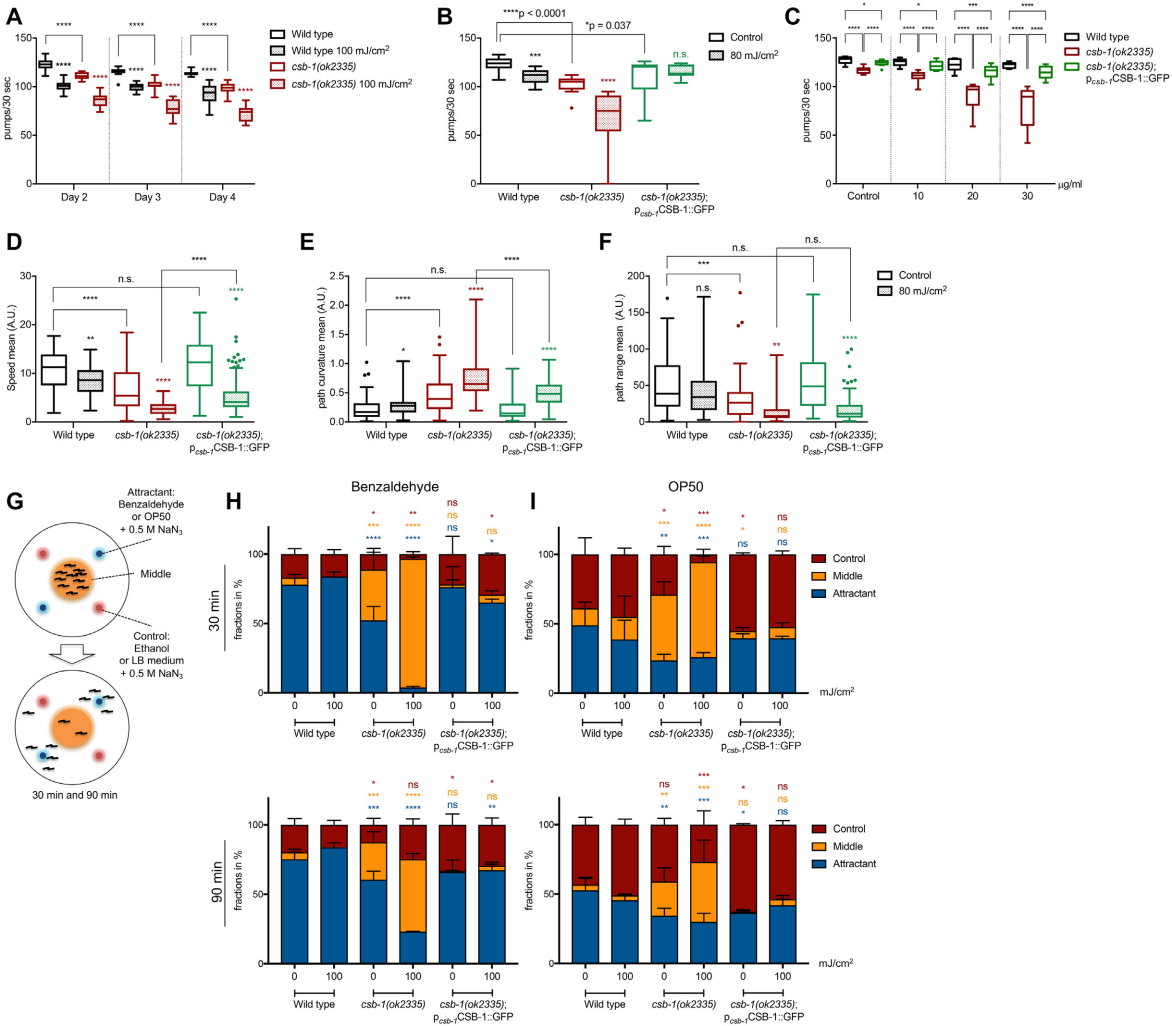
Behavioural changes imply neuronal defects in *csb-1* mutants. (**A**) Pharyngeal pumping in wt and *csb-1* mutants upon UVB irradiation. Nematodes were irradiated at Day 1 of adulthood, and assayed 24 h after irradiation (Day 2), 48 h later (Day 3) and 72 h later (Day 4) (*n* > 15 per group). (**B**) Pharyngeal pumping measured in the wt, the *csb-1* mutant and the *csb-1(ok2335)*;CSB-1::GFP rescue line upon UVB irradiation. Nematodes were irradiated at Day 1 of adulthood, and assayed 48 h after irradiation (*n* > 13 per group). (**C**) Pharyngeal pumping in the wt and *csb-1(ok2335)* (each carrying the p*_mec-4_*GFP neuronal reporter) and the *csb-1(ok2335)*;CSB-1::GFP rescue line upon Illudin M treatment at different concentrations. Whiskers in (A) to (C) show the SD, statistics were computed with the non-parametric Mann-Whitney test with *P < 0.05, **P < 0.01, ***P < 0.001 and ****P < 0.0001 and Tukey outliers. (**D**, **F**) display locomotion features of wt, the *csb-1* mutant and the *csb-1(ok2335)*;p*_csb-1_*CSB-1::GFP rescue line 24 h after UVB irradiation, with (D) mean speed, (E) mean path curvature, and (F) mean path range (in A.U.) (*n* ≥ 60). Significance is measured via the Mann-Whitney test with *P < 0.05, **P < 0.01, ***P < 0.001 and ****P < 0.0001, and Tukey outliers are shown as dots. Statistical significance in (A) to (F) without specific indication always refers to the colour-matched untreated control. (**G**) Experiment scheme of the chemotaxis assay (see Materials and Methods). (**H**) and (**I**) are chemotaxis assays in the wt, the *csb-1* mutant and the *csb-1(ok2335)*;p*_csb-1_*CSB-1::GFP rescue line, quantified after 30 and 90 min, while (H) uses benzaldehyde, and (I) OP50 *E. coli* as attractant, respectively. Significance between zone-specific localization compared to the wt is measured by using the Welch's *t*-test with *P < 0.05, **P < 0.01, ***P < 0.001 and ****P < 0.0001.

About 200 Day 1-adult worms per strain and condition were treated with UVB (100 mJ/cm^2^), or mock-treated, respectively. The next Day (24 h post-UVB irradiation) the animals were washed three times with M9 to remove residual bacteria. Chemotaxis assay plates were freshly prepared 15 min prior to the experiment as previously described (26). Briefly, the chemoattractant 1 μl of benzaldehyde (Sigma-Aldrich, 1:200 dilution in Ethanol) was spotted on empty 60 mm NGM agar plates in equidistant angles, ∼2.5 cm from the center (neutral zone), as indicated in Figure 1C. Ethanol served as control. For OP50 *Escherichia coli* bacteria attraction, a saturated over-night culture was prepared and 10× concentrated. 1 μl of OP50 was spotted on NGM agar plates, while LB medium served as control. To each spot 1 μl of 1 M NaN_3_ (sodium azide, Sigma-Aldrich) was added. *C. elegans* were concentrated in a volume of 20 μl and placed in the center of the assay plates. Worm distribution on the plates was observed 30 and 90 min after assay start.

### Pharyngeal pumping assay

*Caenorhabditis elegans* at Day 1 of adulthood were irradiated with different UVB doses (as indicated), and their pharyngeal pumping rates were accessed as the number of pumps in 30 s, on the day of irradiation, and during the two following days. Measurements were taken at a Zeiss Axio Zoom V.16.

### Oxygen consumption assay

Oxygen consumption was assessed using the Agilent Seahorse XFe96 Analyzer machine and the software Seahorse Wave adopted from (27). Worms were irradiated with UVB radiation at different doses during the early L4 larval stage, and were placed for measurements in 96-well plates in groups of 10 worms per well at the indicated time-points. The calibration plate was prepared the night before the experiment, and kept at 37°C overnight, as described by the manufacturer. On the Day of the assay, the calibration plate was allowed to reach room temperature before being used to calibrate the machine. After calibration, the plate with worms was added and the basal oxygen consumption rate (OCR) was measured 10 times.

### Neuronal defect scoring

Using a confocal Zeiss AxioImager M1 microscope at 40× magnification, the neuronal defect classifications were observed and scored. Fifty worms carrying the *zdls5[mec-4::GFP+lin-15(+)]* I. reporter transgene that were UVB treated at Day 1 of adulthood were placed on a slide with 2% agar pad, in 20 μl levamisole (Sigma Aldrich, 5 mM in water). Axonal beading severity was quantified in the same way. Different categories of severity of beading are presented according to the number of beads counted per animal. Time-lapse images of axonal beading progressing into degeneration were taken on Day 8 after UVB treatment of adult worms, using a similar method and microscope as described above, however with 5% agar pads and 20 μl of nanobeads (Polysciences, Inc., 2.5% by volume, 0.1 μm diameter) to ensure the animal did not dry out.

### Gentle touch sensitivity assay

Worms were synchronized via sodium hypochlorite treatment and grown into Day 1 of adulthood, to be UVB-treated. For the measurements at the indicated time-points, the animals were stroked gently across their body with an eyelash hair, at the head and behind the pharynx to stimulate the ALM neurons, and close to the tail and the anus to stimulate the PLM neurons. The following were classified as a sensitivity to the stimulus: if the animal was still but moved after touch, if the animal was moving but stopped after touch, and if the animal was moving and changed directions after touch (28).

### Neuronal degeneration scoring

Neuronal degeneration was scored using the *zdls5[mec-4::GFP+lin-15(+)]I* reporter line and a Zeiss AxioImager M1 microscope at 63× magnification. Thirty worms that were UVB treated at early L4 stage were placed on a slide with 2% agar pad in 20 μl of levamisole (Sigma Aldrich, 5 mM in water) for immobilization. Beading axons were localized and closely investigated for the absence of axons between the beads, indicating neuronal breakage. For timelapses the animals were immobilized in 20 μl of nanobeads (Polysciences, Inc., 2.5% by volume, 0.1 μm diameter) as described previously (29).

### Illudin M treatment

Illudin M (Cayman Chemical) was dissolved in dimethylsulfoxide (DMSO) to a stock concentration of 0.25 mg/ml. For the treatment, worms were synchronized via hypochloride treatment and grown into the L4 stage on OP50-seeded NGM plates. The animals were washed three times with M9 and then treated for 16 h in 2 ml K-medium (2.36 g KCl, 3 g NaCl in 1 l ddH_2_O, autoclaved) containing cholesterol (5 μg/ml), heat-inactivated OP50, and Illudin M in the indicated concentrations. We added 0.075% DMSO to the control, which corresponds to the 30 μg/ml Illudin M treatment. After the treatment, the samples were washed three times with M9 and the worms were seeded to OP50-seeded NGM plates. Upon a recovery phase of 2 h, pharyngeal pumping rates were determined. 24 h after, the neuronal network was closely examined for neuronal defects, as described above.

### Mitochondrial mass assays

For the measurements of mitochondrial mass, the animals with the reporter lines *zcls14[myo-3::GFP(mit)]* and *zcls17[ges-1::GFP(mit)]* were grown synchronized until the early L4 larval stage and treated with UVB. At the different time points assayed, the animals were washed off the plates with M9 medium, washed once with M9 and pelleted at 1300 rpm for 1 min. The supernatant was removed to approx. 20 μl and 2 μl of 50 mM levamisole were added before mounting the animals on 2% agarose pads for imaging at a Zeiss AxioImager M1 microscope with a 5× lens. For the large particle flow measurements the animals were transferred to 50 ml falcons to be quantified in the Biosorter (Union Biometrica BioSorter with FlowPilot Sotware).

### TMRE assay

For the TMRE staining experiments, *C. elegans* were grown until the early L4 larval stage and UVB-treated. TMRE staining at the indicated time points was done on NGM agar plates at a concentration of 30 μM dissolved with heat inactivated OP50 (30 min, 65°C). Once the plates were dry, animals were added via picking and left to take up the dye for 2 h. After staining, the animals were washed with M9 and moved to NGM plates spread with heat inactivated OP50 to avoid active bacteria from taking up the dye and staining the intestines of the worm. After 1 h crawling on the plates without the dye, the animals were washed and prepared for imaging as described in the mitochondrial mass assay. For large particle flow quantification the worms were washed into 50 ml falcons and fluorescence intensity levels were measured via the biosorter.

### Mitochondrial network analysis

Using the *zcls14[myo-3::GFP(mit)]* reporter line, the mitochondrial network was examined by observing the muscle cell number 18. Synchronized early L4 animals were treated with UVB radiation, and at the different time points, 30 animals per condition were scored using a confocal Zeiss AxioImager M1 microscope at ×63 magnification. Worms were mounted onto slides with 2% agar, and immobilized in 20 μl of nanobeads (Polysciences, Inc., 2.5% by volume, 0.1 μm diameter).

### Statistical analysis

Statistical analyses and graphing were carried out using the Prism software package (GraphPad Software Inc., San Diego, USA) and the statistical computing language R (http://www.R-project.org). The statistical tests applied for each experiment are presented in the figure legends.

## RESULTS

### Somatic tissue functionality is reduced in *csb-1* mutant animals

CS patients show a number of behavioural, cognitive and perceptual abnormalities, including ocular abnormalities, sensorineural hearing loss and disturbed gait (4). We used *csb-1* deficient *C. elegans* to assess possible behavioural defects with or without UV treatment of post-mitotic, somatic tissues in adult animals. Pharyngeal pumping rates are a sensitive parameter of the functionality of a somatic tissue and decline rapidly in completely NER-deficient *xpa-1* mutant animals following UVB treatment (10,15). *csb-1* mutant animals show decreased pharyngeal pumping rates compared to wildtype (wt) animals at Day 2 of adulthood, which was accentuated with UV irradiation and progressed over the first 4 days of adulthood (Figure 1A). To ensure that this result was in fact due to the loss of CSB-1, we generated a genetic rescue transgenic strain that ectopically expresses the wt CSB-1 protein under the control of the *csb-1* promoter in the *csb-1* mutant background (*csb-1(ok2335)*;p*_csb-1_*CSB-1::GFP). This genetic rescue strain showed a significant alleviation of the pumping defect. The UV sensitivity of *csb-1* mutants was completely restored in the rescue line (Figure 1B). To further validate whether the declining tissue functionality was caused by transcription-blocking lesions, we next assessed pharyngeal pumping in the animals treated with Illudin M. This sesquiterpene from the fungus *O. illudens* inflicts transcription-blocking DNA lesions and is highly cytotoxic to TC-NER deficient human cells and, when applied during early development, compromises larval growth in TC-NER deficient worms (13,30,31). Illudin M treatment of *csb-1* mutant animals resulted in a dose-dependent decline in pharyngeal pumping that was significantly rescued by transgenic expression of wt CSB-1::GFP (Figure 1C).

We extended our analysis to locomotion functions and found that the mean speed of adult *csb-1* mutants was significantly reduced (Figure 1D). At the same time, the sinusoidal path curvature that is characteristic for *C. elegans* movement was abnormally enhanced, resulting in a shortened mean path range (Figure 1E, F). Locomotion dysfunction was significantly increased upon exposure to UV, which was partially suppressed in the CSB-1::GFP rescue line (Figure 1D–F). Similarly, Illudin M treatment caused a reduction in mean speed that was strongly exacerbated in *csb-1* mutants and in turn significantly alleviated by the transgenic expression of the CSB-1::GFP (Supplementary Figure S1). To further address the neuronal functionality, we assessed attraction behaviour to volatile chemicals, which is mediated by a subset of chemosensory neurons in *C. elegans* (32). We used a chemotaxis assay to test the attraction of *csb-1* mutants to benzaldehyde, or OP50 *E. coli* bacteria, respectively (Figure 1G) (26). While most wt animals localised to the chemoattractants within 30 min, *csb-1* mutants resided in the neutral areas of the plate for extended time periods, indicating a loss of chemosensory function, a phenotype that persisted after 90 min. Chemoattraction was restored in the CSB-1::GFP rescue line. Pre-treatment with UV robustly aggravated loss of chemosensation specifically in *csb-1* mutants (Figure 1H, I). Together, these results show that CSB-1 deficient *C. elegans* display behavioural alterations, which are indicative of neuronal dysfunctions.

### *csb-1* mutants have gentle touch mechanosensory neuronal defects

**Figure 2.**
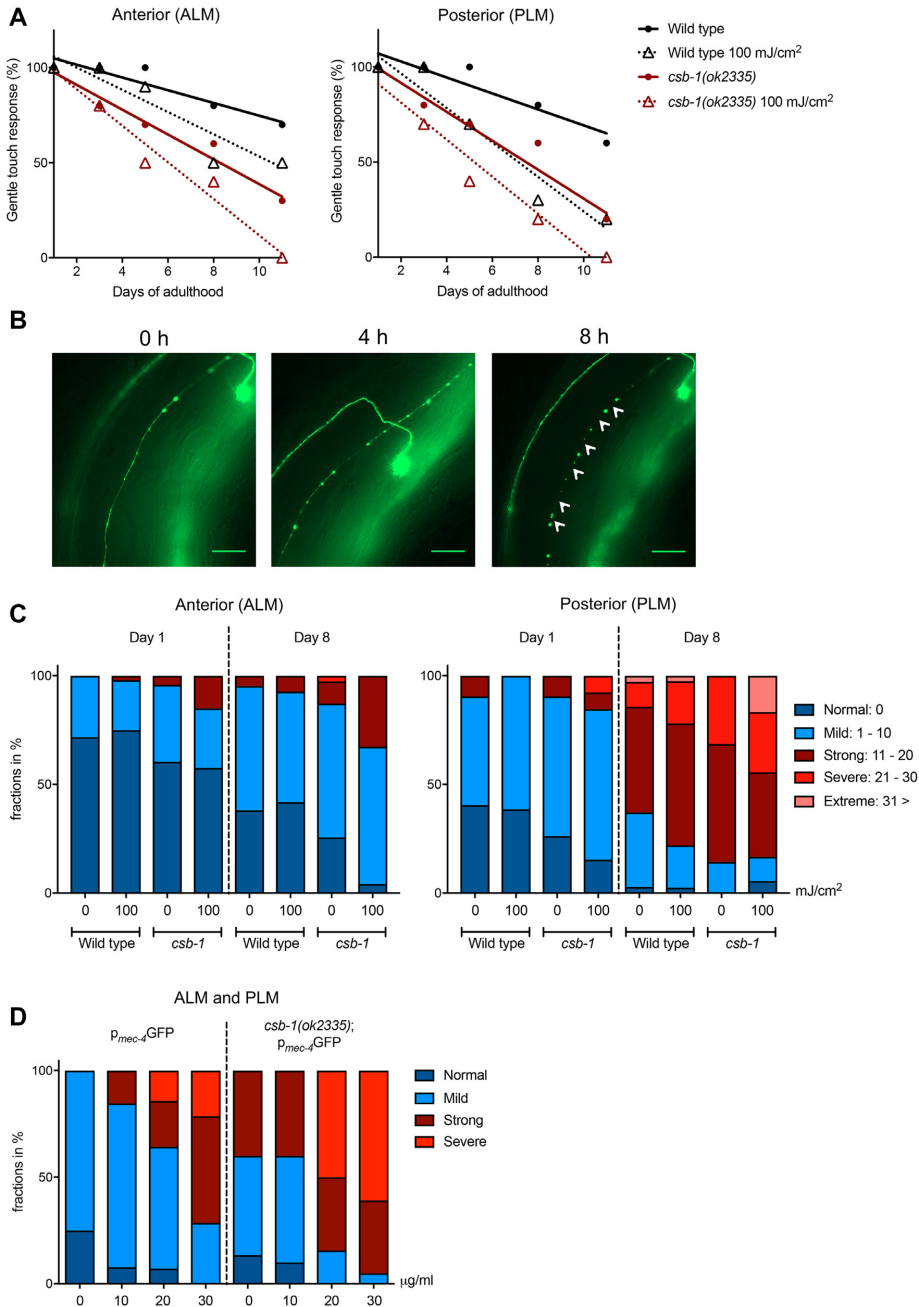
Loss of neuronal integrity is accentuated in *csb-1* mutants. (**A**) Gentle touch response assayed in the anterior part of the animal (ALM neurons), or the posterior (PLM neurons), respectively. Animals were treated with UVB at young adult stage, and measured on the Day of UV radiation (Day 1), and on the Days 3, 5, 8 and 11 thereafter. Plot shows nonlinear regression (curve fit) line. (**B**) Representative images of neuronal beading in a time-dependent manner in animals expressing p*_mec-4_*GFP. Gentle touch mechanosensory neurons were irradiated at Day 1 of adulthood and neuronal beading was observed for morphological changes at Day 8 (time: 0) after treatment. The first timepoint shows a neuron with beading. With time, the beading becomes more severe, until it reaches a stage with breakages along the axon resulting in degeneration, which is indicated by white arrows. Scale bars are 25 μm. (**C**) Quantification of degeneration in anterior (ALM) and posterior (PLM) mechanosensory neurons. Animals were treated at young adult stage, and measured on the Day of treatment (Day 1) and 7 Days after (Day 8). Degeneration is measured by quantifying the severity of beading per animal. Statistical analysis is available in Supplementary Table S3. (**D**) Quantification of neuronal beading along ALM and PLM axons in animals 24 h post-treatment with different concentrations of Illudin M.

CS patients show progressive neurological dysfunction, with abnormal myelin, brain atrophy and microcephaly being key features (1). To investigate whether *C. elegans* deficient for CSB-1 show signs of neurodegeneration, we examined the gentle touch response that is mediated by the mechanosensory neurons, posterior lateral microtubule cells (PLM), anterior lateral microtubule cells (ALM), and posterior ventral microtubule cell (PVM) (18). The integrity of those neurons can be followed in live worms by using the *zdls5[mec-4::GFP+lin-15(+)]* I. reporter line that expresses GFP under the control of the neuron-specific *mec-4* promoter (33). To assess neuronal functionality we performed an age-dependent longitudinal study, testing the sensitivity of the animals to gentle touch triggered by strokes with an eyelash hair to stimulate posterior or anterior neurons, respectively. Compared to the wt animals, *csb-1* mutants showed an accelerated age-dependent loss of touch sensitivity. UV treatment exacerbated the age-dependent touch insensitivity indicating that DNA damage can promote the loss of neuronal functionality (Figure 2A, statistics in Supplementary Table S2A, B).

In order to examine why the sensitivity to gentle mechanosensory stimuli was lower in the *csb-1* mutant strain, we questioned whether the animals were losing neuronal integrity. Using the double mutant *zdls5[mec-4::GFP+lin-15(+)]I;csb-1(ok2335)*, we scored for neuronal defects that previously have been characterised in an age-dependent analysis of neuronal decline (20). We identified a range of abnormalities, including axonal beading, axonal degeneration, axonal disorganization (zig-zag-like structures), axonal branching, protrusions emerging from the neuronal cell body, and bridging of axons (Supplementary Figure S2A). Axonal beading was the most abundant structural aberration in wt and *csb-1* mutants, which prompted us to quantify this phenotype. We hypothesized that the axonal beading was preceding the axonal degeneration. To examine this, we carried out a time-lapse study, and confirmed that, over the course of several hours, an axon with beads can increase in severity concomitantly with a thinning of the axon, until it leads to breakages in the axon, i.e. neurodegeneration (Figure 2B). We then counted the number of beads in ALM and PLM neurons and categorized the animals into absent, mild, strong, severe and extreme. Consistent with the functional degeneration observed in Figure 2A, the *csb-1* mutant strain showed a stronger beading phenotype compared to wt animals, which was enhanced upon UV irradiation (Figure 2C, statistics in Supplementary Table S3A, B). Further, we quantified axonal degeneration indicated by the absence of axons between beads in PLM neurons and found a significant increase in adult *csb-1* mutants (Supplementary Figure S2B). Similar to UV irradiation, treatment with Illudin M significantly enhanced the neuronal beading phenotype in the wt background, and, even more severely, in CSB-1 deficient animals (Figure 2D).

### Loss of *csb-1* causes an accumulation of dysfunctional mitochondria

**Figure 3.**
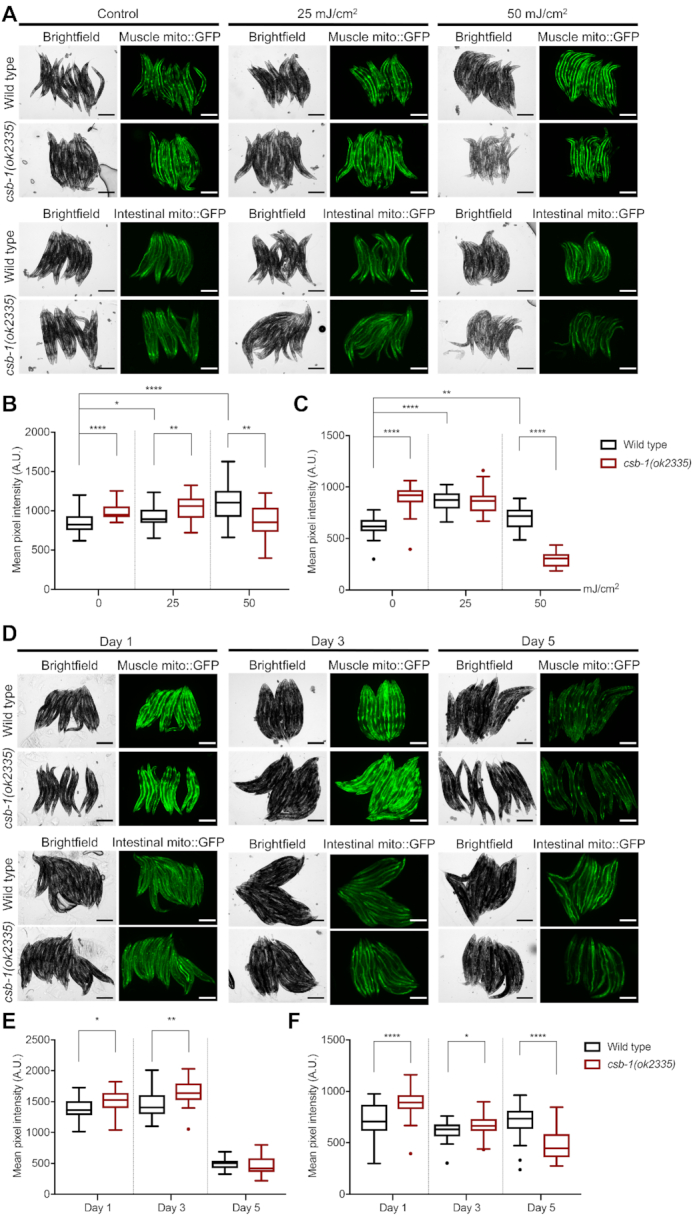
Mitochondrial mass accumulates in *csb-1* mutants. (**A**) Representative images of animals expressing muscular (p*_myo-3_*mito::GFP, upper panel) or intestinal (p*_ges-1_*mito::GFP, lower panel) mitochondrial localized GFP upon UVB irradiation. The brightfield images and the corresponding fluorescence images are shown. (**B**) Quantification of p*_myo-3_*mito::GFP. (**C**) Quantification of p*_ges-1_*mito::GFP. (n = 25 animals). Whiskers in (B) and (C) show the SD. (n = 25 animals). Statistics were computed with the non-parametric Mann–Whitney test with *P < 0.05, **P < 0.01, ***P < 0.001 and ****P < 0.0001 and Tukey outliers are shown. (**D**) Representative images of animals expressing muscular (p*_myo-3_*mito::GFP, upper panel) or intestinal (p*_ges-1_*mito::GFP, lower panel) GFP in mitochondria during adult ageing. The brightfield images and the corresponding fluorescence images are shown. (**E**) Quantification of p*_myo-3_*mito::GFP. (**F**) Quantification of p*_ges-1_*mito::GFP. Error bars in (E) and (F) show the SD (*n* = 25 animals). Statistics were computed with the non-parametric Mann–Whitney test with *P < 0.05, **P < 0.01, ***P < 0.001 and ****P < 0.0001 and Tukey outliers are shown. Size bars in (A) and (D) are 250 μm.

CS patients display a variety of symptoms that can also be found in mitochondrial diseases and the neurological defects in CS might be connected to mitochondrial dysfunction (1,34). In human cell lines, CSB-deficiency causes the accumulation of damaged mitochondria paralleled by altered mitochondrial function (6). We employed the strains *zcls14[myo-3::GFP(mit)]* and *zcls17[ges-1::GFP(mit)]* that express GFP with a mitochondrial targeting sequence under the control of a muscle or intestine specific promoter, respectively, to visualize mitochondrial content in *C. elegans* tissues (35). We found that mitochondrial GFP levels in *csb-1* mutants were significantly higher as compared to wt worms, indicating an increase in mitochondrial mass (Figure 3). However, exposure to UV (25 and 50 mJ/cm^2^) caused an increase of mitochondria-targeted GFP in wt animals, while *csb-1* mutants showed significantly less mitochondrial mass at 50 mJ/cm^2^ UVB (Figure 3A-C). We validated these results for the *zcls17[ges-1::GFP(mit)]* strain by applying large-particle flow cytometry for quantitative GFP measurements in whole worms (Supplementary Figure S3A). An age-dependent analysis revealed that mitochondrial GFP levels were significantly elevated in *csb-1* mutants at Day 1 and Day 3 in adulthood compared to the wt, while at Day 5 the situation reversed for the intestinal mito::GFP reporter (Figure 3D–F).

**Figure 4.**
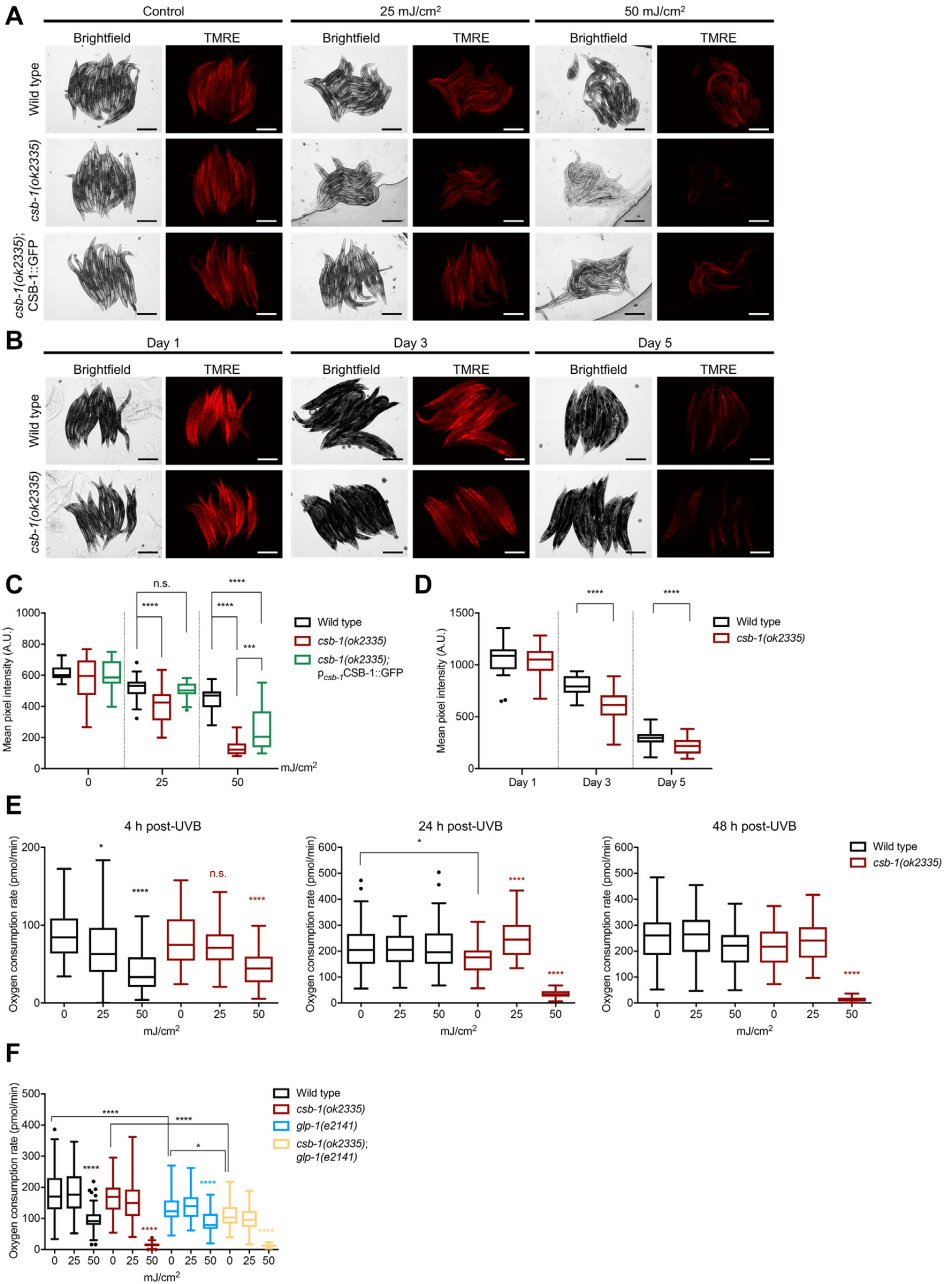
*Csb-1* mutants display mitochondrial damage upon UVB and during ageing. (**A**) Representative images of animals stained with the mitochondrial dye TMRE upon UVB irradiation or (**B**) during ageing. Images show the brightfield and the corresponding red fluorescence signal of TMRE. (**C**) Quantification of data represented in (A) comparing wt, *csb-1(ok2335)* and the CSB-1::GFP rescue (*n* = 25). (**D**) Quantification of data represented in (B) (*n* = 25). (**E**) Oxygen consumption rates of *csb-1* mutants 4, 24 and 48 h after UVB irradiation (*n* > 50 per group, >5 wells with biological replicates, measured 10 times). Results are presented per wells containing 10 animals each. (**F**) Oxygen consumption rate of *glp-1;csb-1* double-mutants after UVB irradiation (*n* > 50 per group, >5 wells with biological replicates, measured 10 times). Panels (C) to (F) show boxplot whiskers representing the SD with Tukey outliers indicated as dots. Statistical significance without specific indication always refers to the colour-matched untreated control. Statistics were computed with the Mann–Whitney test while *P < 0.05, **P < 0.01, ***P < 0.001 and ****P < 0.0001. Size bars in (A) and (B) are 250 μm.

The higher levels of mitochondrial content in both tissue-specific mitochondrial GFP transgenic lines in the *csb-1* mutant strain suggest that the turnover of mitochondria might be compromised, indicative of the accumulation of damaged mitochondria. To address this question, we tested the levels of active mitochondria via staining with tetramethylrhodamine ethyl ester (TMRE), a positively charged, cell permeant dye, which accumulates in functional mitochondria due to their negative membrane potential. Despite their higher mitochondrial content, *csb-1* mutants at Day 1 of adulthood showed similar levels of mitochondrial activity (Figure 4A-D, control). However, after treatment with UV, the *csb-1* mutants showed a significantly lower level of active mitochondria, which was rescued by re-expression of wt CSB-1 in the CSB-1::GFP rescue line (Figure 4A, C). An analysis at different time-points during adulthood revealed that *csb-1* mutants contain less active mitochondria at Day 3 and Day 5 of adulthood (Figure 4B, D). We further validated the TMRE quantification of intact mitochondrial levels after UVB irradiation by large-particle flow cytometry (Supplementary Figure S3B).

Mitochondrial dysfunction has been postulated as an important pathomechanism of CS (6,34). Therefore, we examined oxygen consumption rates (OCR) as a parameter for respiratory chain activity. Under unperturbed conditions, *csb-1* mutants showed similar OCR as wt animals. When irradiated at the L4 stage, wt and *csb-1* mutant animals displayed a dose-dependent reduction in the oxygen consumption after 4 h. While wt animals recovered within 24 h, the OCR dramatically decreased in *csb-1* mutants upon 50 mJ/cm² of UVB and failed to recover. Upon 25 mJ/cm^2^ we observed a slight transient increase by 24 h in *csb-1* mutants pointing to a compensatory OCR increase (Figure 4E).

As CSB-1-mediated TC-NER is specifically required in somatic tissues but not the germline where GG-NER predominates (14,15), we wished to decouple any potential contribution by mitochondria in the germline to the OCR measurements. To verify that such differences reflected OCR in somatic tissues and were not due to effects of UV on the germline we employed *glp-1;csb-1* double mutants. The GLP-1 protein is part of the Notch family of transmembrane receptor proteins and is involved in the regulation of mitotic germ cell divisions. The *glp-1(e2141)* loss-of-function allele is a temperature sensitive mutant, which does not develop a germline when grown at 25°C (36). We hypothesized that the strains in the *glp-1* background would have a generally lower OCR due to the absence of an energy-demanding organ such as the germline. Indeed, *glp-1* mutants showed a lower level of OCR compared to the wt, and the *glp-1;csb-1* double mutant displayed a lower level of OCR compared to *glp-1* or *csb-1* single mutants, respectively, while maintaining the accentuated reduction in response to 50 mJ/cm² in the *csb-1* mutant strain (Figure 4F).

Taken together, these results suggest that the increased mitochondrial mass in *csb-1* mutants compensates for their relative dysfunctionality, thus maintaining respiratory activity. Only at a higher DNA damage load (50 mJ/cm²) respiratory activity cannot be maintained.

### *Csb-1* mutants show a hyperfused mitochondrial network

**Figure 5.**
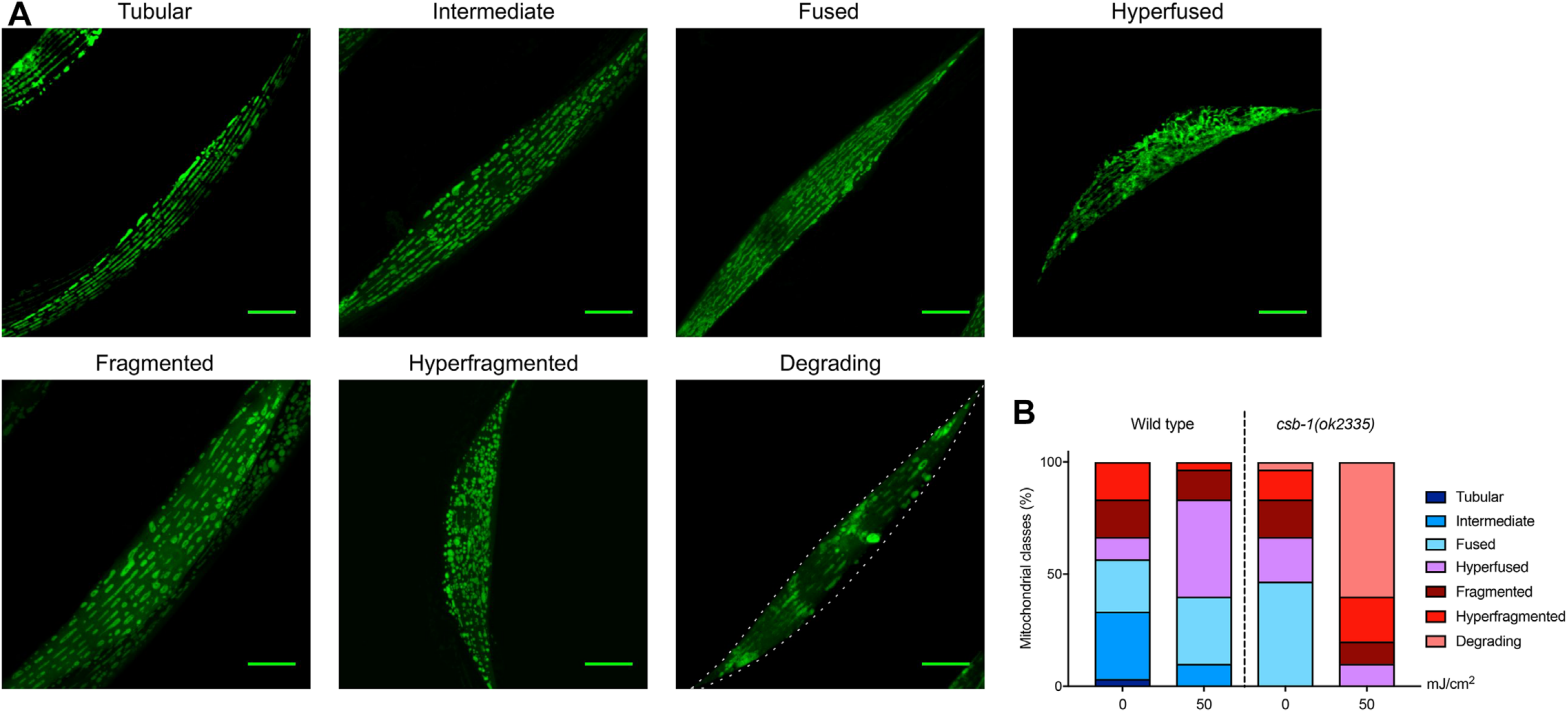
Mitochondrial structure aberrations in *csb-1* mutants. (**A**) Representative confocal images of the different mitochondrial morphology classes: ‘Tubular’ and ‘Intermediate’ mitochondrial classifications describe the unstressed mitochondrial status. The classifications ‘Fused’ and ‘Hyperfused’ belong to the mechanism mitochondria undergo in order to recover from a mild stress. The classifications ‘Fragmented’, ‘Hyperfragmented’ and ‘Degrading’ belong to the path mitochondria follow in response to acute stress. The dotted line represents the outline of the cell. Scale bars are 15 μm. (**B**) Quantification of mitochondrial classes in wt and the *csb-1* mutant. Animals were treated at L4 stage, and measured 24 h after.

The observed accumulation of dysfunctional mitochondria prompted us to further investigate the mitochondrial network structure. CSB-deficient human cell lines display a higher variation in mitochondrial width, indicative of greater mitochondrial heterogeneity (6). Mitochondrial dynamics play a role in this diversity with fission and fusion as key modulators of the organelle structure, which can be activated or inhibited by different stressors. While mild stress often leads to hyperfusion of mitochondria as a first line of defence, a more severe stress leads to hyperfragmentation and mitophagy in order to segregate and eliminate dysfunctional mitochondria from the network (37). Indeed, when treating the wt and *csb-1* mutants with UV radiation, different types of mitochondrial structure were observed, which we categorized into tubular, intermediate, fused, hyperfused, fragmented, hyperfragmented, and degrading (Figure 5A). These classifications were used to score the phenotypes of the mitochondrial network structure in wt and *csb-1* mutant worms. In wt animals about 30% of the cells had mitochondria that displayed tubular and intermediate shapes. Upon UV exposure, these categories were severely diminished (Figure 5B, left-side, statistics in Supplementary Table S4). The majority of *csb-1* mutants contained fused or hyperfused mitochondria, while tubular or intermediate organelles were absent. Once stressed with UV, most of the animals showed degrading or fragmented mitochondria (Figure 5B, right-side, Supplementary Table S4). These data suggest that endogenous DNA lesions that remain unrepaired due to the *csb-1* mutation result in mitochondrial damage, which is greatly aggravated by exogenous DNA damage conferred by UV irradiation.

Taken together, *csb-1* mutant worms display multiple mitochondrial defects and compromised respiratory activity, concomitant with neuronal degeneration. Consistent with such abnormalities resulting from the defects in repairing endogenous DNA damage, both mitochondrial defects and neurodegenerative phenotypes are greatly accentuated by UV irradiation.

## DISCUSSION

Here, we establish phenotypic parallels between human CS and *C. elegans* carrying a *csb-1* mutation that allows investigating the underlying role of DNA damage in accelerating neuronal degeneration *in vivo*. We find that *C. elegans csb-1* mutants display a range of phenotypes that reconstitute human CS pathologies (summarized in Table 1). We demonstrate that *csb-1* mutants show decreased tissue functionality as assayed by the highly sensitive assessment of pharyngeal pumping activity. Similarly, human patients present reduced tissue functionality and feeding (3,38–40). In contrast, *Csb* mutant mice show increased food consumption and no apparent changes in overall tissue functionality (6). We determine that *csb-1* mutant worms have defects in chemotaxis, sinusoidal locomotion and touch sensitivity pointing to a loss of neuronal functionality. These findings are reminiscent of CS symptoms in human patients who suffer from ataxia, spasticity and a demyelinating neuropathy, resulting in impaired ambulation and reflexes, as well as delayed motor and cognitive development (1,39). In relation to the functional neurological decline, we visualised progressive neurodegeneration of mechanosensory neurons, which play a multimodal role in touch sensation and locomotion control of *C. elegans* (41). Although substantially varying across cases, brain atrophy, neuronal loss and axonal degeneration commonly occur in CS patients (42). Contrariwise, *Csb* mutant mice are lacking concrete evidence for neuronal defects or neurodegeneration (7,43), except for a loss of spiral gangliocytes in the inner ear of old *Csb* mutant mice (6). Because of the limited phenotypic consequences of a *Csb* mutation in mice (see Table 1), the effect of the *CSB* deficiency on neuronal functionality has not been systematically studied *in vivo*. Therefore, we characterized *csb-1* mutant *C. elegans* as CS model for studying morphological and molecular changes in neurons and their phenotypic consequences on the animals’ behaviour. Consistent with the requirement of CSB to remove UV-induced lesions in actively transcribed genes in cultured, terminally differentiated human neurons (44), we observed that neuronal defects and behavioural decline were enhanced upon UV irradiation in wt animals and, more severely, in *csb-1* mutant worms. Similarly to UV irradiation, treatment with Illudin M, which induces transcription-blocking lesions (30), accelerates the functional decline and neurodegeneration in *csb-1* mutants. These results pinpoint the causal effect of DNA lesions that obstruct transcription elongation in triggering neurodegeneration. It will be highly interesting to further evaluate the causal contribution of transcription-blocking lesions in neurodegenerative disorders occurring during normal ageing in humans. To this end the nematode might serve as instructive *in vivo* model of neurodegeneration because in contrast to *in vitro* studies in neuronal cultures, the *C. elegans* CS model allows the investigation of the nervous system in a whole-organism context during development and throughout the course of ageing.

**Table 1.** Comparison of characteristics of CSB-deficient models. N/A refers to phenotypes that have not been yet investigated

Feature	Description	Human	*Mus musculus*	*C. elegans*
UV sensitivity	Development	X	X	X
	Post-mitotic	X	X	X
Neuronal	Degeneration	X	(X)	X
	Functional deficit	X	-	X
Mitochondria	Dysfunction	X	X	X
	Structure	N/A	X	X
Healthspan	Tissue functionality	X	-	X
Fertility	Viable offspring	-	X	X
Feeding	Difficulty	X	-	X
Cancer	Absence	X	X	N/A
Muscle functionality	Decreased	X	-	X
Development	Delay	X	-	X*
Body size	Reduction	X	-	X*

*This feature in worms has been observed only upon UV irradiation.

Given that CS is a progeroid disease, insights into the consequences of *CSB* deficiency might further our understanding of the ageing process. In the *C. elegans* CS model, we show a progressive, age-dependent decline in pharyngeal pumping and mechanosensation paired with a gradual loss of neuronal integrity. Despite these functional defects, *csb-1* mutant worms have a normal lifespan that is reduced upon UV-induced DNA damage (13,45). The progressive loss of neuronal integrity in *csb-1* mutants was characterized by an early-onset of increased beading that precedes axonal degeneration. We also detected aberrant neurite morphology, bubble-like lesions (bridging) and neurite sprouting (branching) along the axons of mechanosensory neurons; features that naturally appear during advanced age in the *C. elegans* neuronal system (20). Neuronal maintenance in ageing is governed by the Jun kinase (JNK-1) and insulin signalling via DAF-16/FOXO (20,46,47), both of which have been implicated in the DNA damage response (DDR; (15,48). We previously showed that the activation of the transcription factor DAF-16 upon UV-induced DNA damage promotes developmental growth and lifespan in *csb-1* mutants thus counteracting the consequences of unrepaired DNA damage (13,15). Curiously, age-dependent defects in posterior touch neurons (PLM) are significantly more severe as compared to the anterior (ALM), which might rear from the ability of touch response neurons to differentially adapt to environmental signals that modulate sensory output: While ALMs are capable to respond to insulin-peptides that converge on AKT kinases (AKT-1) and DAF-16/FOXO to mount a stress-induced sensory-adaptation, PLMs are known to lack this level of modulation (49,50). Similarly, the DDR could be differentially regulated in specific neuronal subsets.

What homeostatic processes might be perturbed in *csb-1* mutants that could impact neuronal integrity? Following this intriguing question, we have characterized alterations in mitochondrial morphology in our CS model. Mitochondrial decline is a hallmark of ageing, and mitochondrial homeostasis is essential for neuronal maintenance (51,52). Cells of *Csb* deficient mice accumulate damaged mitochondria, which might be explained by a defect of mitochondrial clearance via autophagy (34). We demonstrate that the lack of the CSB-1 protein leads to the accumulation of mitochondria in muscles and the intestine, while mitochondrial activity throughout tissues in the animal is reduced. Our data suggest that these events are unfolding organism-wide rather than tissue-specific. Further, we detect expansion of the mitochondrial mass and a hyperfused mitochondrial network, while respiratory activity is largely maintained. Our results on mitochondrial morphology changes are consistent with previous findings in *C. elegans* mutants for the *csa-1* or *csb-1* genes (53). Upon exogenous DNA damage, however, the mitochondria fragment and *csb-1* mutants show a strong dampening of oxygen consumption indicative of mitochondrial dysfunction. These observations suggest that mitochondria compensate for the defect in repairing endogenous DNA lesions in *csb-1* mutants by expansion of the network to maintain respiratory activity. However, this adaptive response might be overwhelmed when exogenous lesions cannot be repaired. It will be highly interesting to better understand the molecular mechanisms of the compensatory mitochondrial stress response and determine its role in maintaining tissue functionality, and more specifically neuronal integrity, amid CS pathologies.

The CS model we establish here for neuronal degeneration and mitochondrial aberrations in *C. elegans csb-1* mutants could fill an important experimental gap between the complex human CS pathologies and the lack of experimentally traceable metazoan *in vivo* models reflecting the phenotypic disease manifestations. The completely mapped neural circuitry of *C. elegans* will aid in characterising the specific neurons or type of neurons that are affected by degeneration in *csb-1* mutants and by DNA damage. This model might also shed light onto the long-standing question why neurons are particularly sensitive to DNA repair defects, given that not only CS but a large number of congenital DNA repair deficiencies are characterized by neurodegeneration (54). Our results indicate that transcription-blocking lesion might have a profound effect on triggering neurodegeneration. It is conceivable that the typically high transcription rates of neurons could make them particularly vulnerable to lesions induced by endogenous genotoxins that result from the high neuronal metabolic activity. The CS nematode model can readily be exploited not only for mechanistic, physiological and genetic studies but also for identifying and characterizing pharmacological interventions that might ameliorate the disbalance of the mitochondrial network and prevent neurodegeneration. The *C. elegans* CS model might thus yield important new insight into the causal role of DNA damage in the age-related progressive loss of neuronal integrity and provide an experimental model for neurodegenerative diseases.

## Supplementary Material

gkaa795_Supplemental_File

## Notes

Present address: Amanda F.C. Lopes, Department of Clinical Neurosciences, School of Clinical Medicine, and Medical Research Council Mitochondrial Biology Unit, University of Cambridge, Cambridge Biomedical Campus, Cambridge CB2 0QQ, UK.
