# Supplementary material for: A C. elegans model for neurodegeneration in Cockayne syndrome
Source: Nucleic Acids Res. 2020 Oct 6;48(19):10973–85. doi: 10.1093/nar/gkaa795 (PMC7641758; doi:10.1093/nar/gkaa795)
Supplement: gkaa795_Supplemental_File [file gkaa795_supplemental_file.pdf]

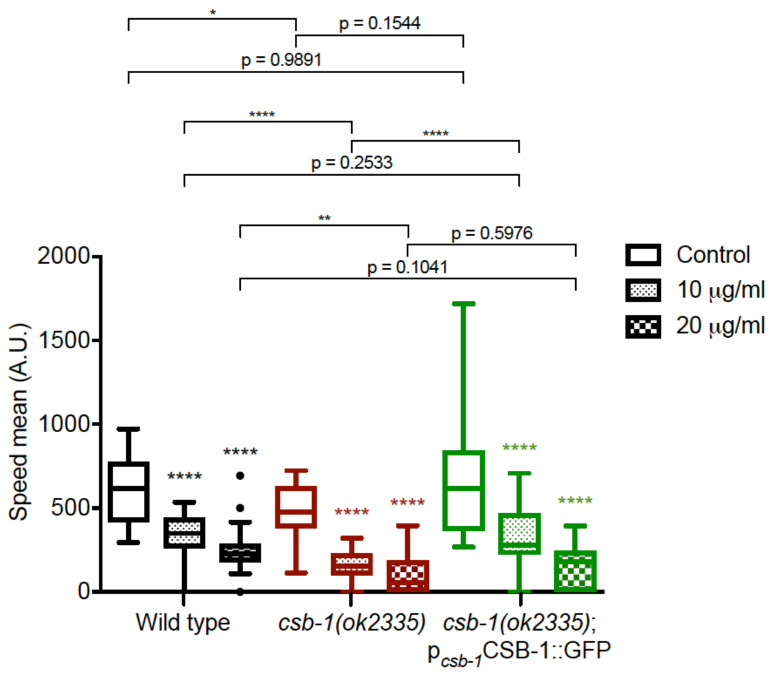

Supplementary Figure 1: Mean speed (in A.U.) of *zdls5*[mec-4::GFP+lin-15(+)] I. (wt), the *csb-1;zdls5*[mec-4::GFP+lin-15(+)] I. (*csb-1*) and the *csb-1(ok2335)*;p$_{csb\text{-}1}$CSB-1::GFP rescue line 24 h after Illudin M treatment (n ≥ 15). Significance is measured via the Mann-Whitney test with *p < 0.05, **p < 0.01, ***p < 0.001, and ****p < 0.0001, and Tukey outliers are shown as dots. Statistical significance without specific indication always refers to the colour-matched untreated control.

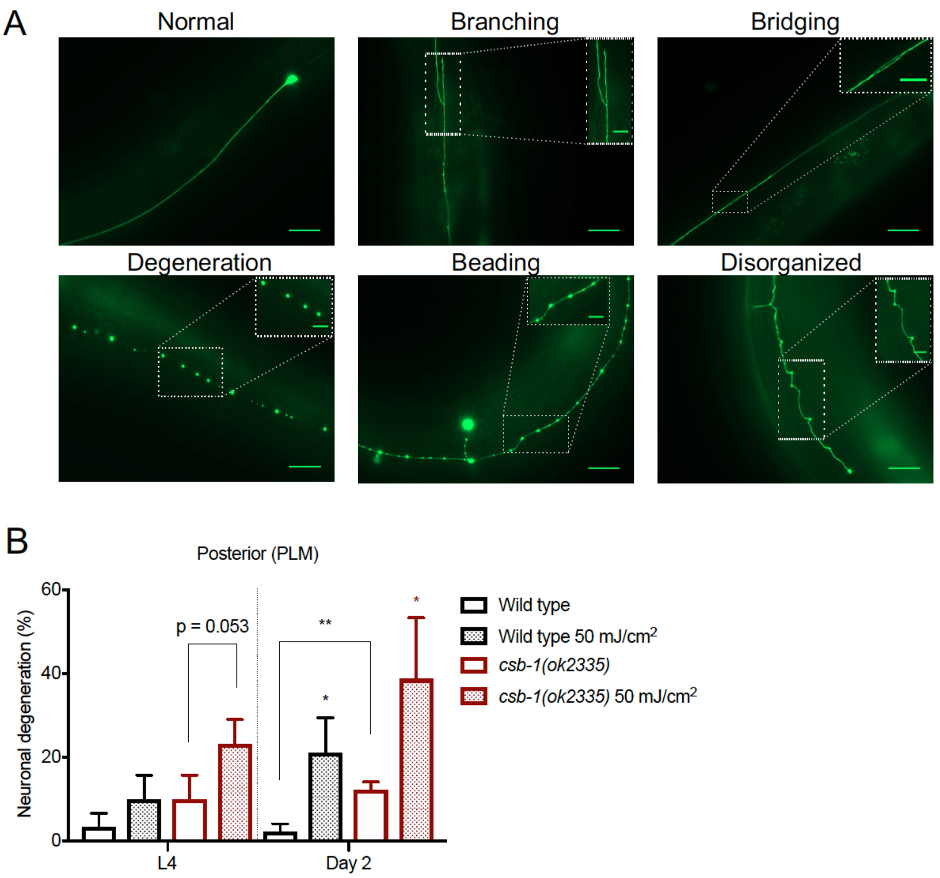

Supplementary Figure 2: (A) Various categories of neuronal aberrations observed in the transgenic strains *zdls5*[mec-4::GFP+lin-15(+)] I. and *zdls5[mec-4::GFP+lin-15(+)]I;csb-1(ok2335)*. We observed axonal branching, axonal bridging (extensions leaving and rejoining onto the main axon), disorganisation (neurons with zig-zag-like patterns), and axonal beading (aggregations along the axon). Degeneration can be seen as aggregates that become disconnected while often still arranged in a line. Scale bars correspond to 25 µm. Zoomed inlay contain scale bars of 10 µm. (B) Quantification of neuronal degeneration in PLM neurons scored as animals showing breakages between beadings along axons. Results of three independent repetitions (each with n = 30) are pooled and analysed with the unpaired t-test with Welch's correction for significance with $p < 0.05$ and $**p < 0.01$.

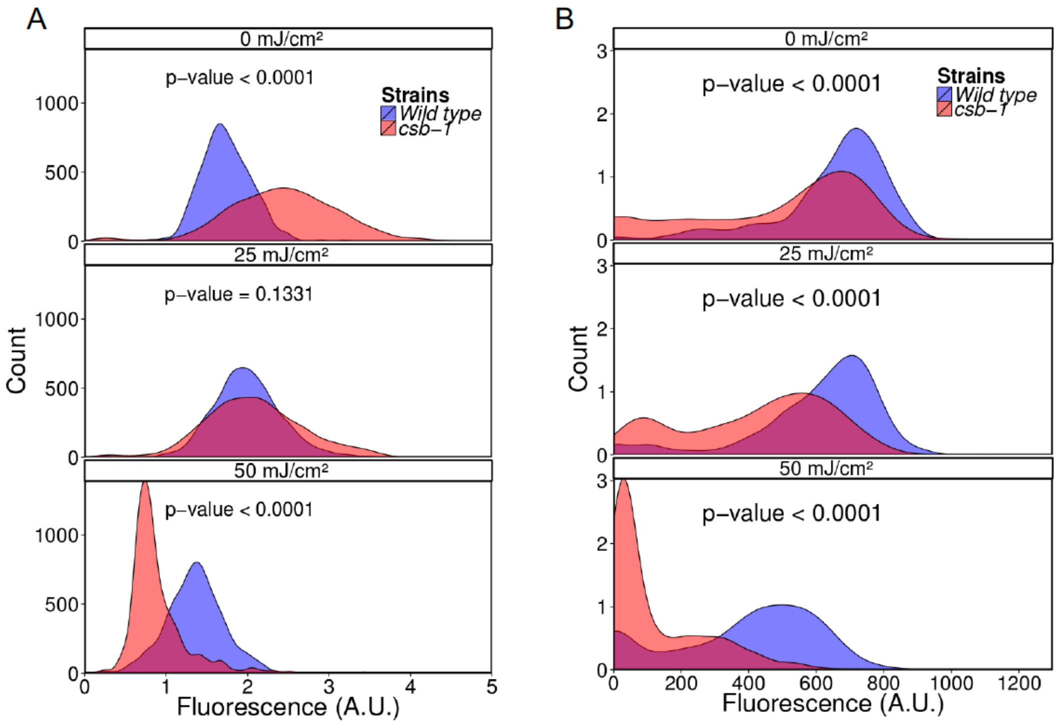

Supplementary Figure 3: (A) Mitochondrial content of intestinal tissues (*zcls17[ges-1::GFP(mit)]*) of *csb-1* mutants 24 h after UVB irradiation. Nematodes were irradiated at early L4 stage and grown at 20 °C. (n>450 per group, triplicate measurements per experiment). (B) TMRE staining to quantify intact mitochondrial levels after UVB irradiation. Animals were stained with TMRE for 1 h before measurement. Fluorescent levels were measured in wt and *csb-1* mutants 24 h after UVB irradiation. Nematodes were irradiated at early L4 stage and grown at 20 °C (n>650 per group). Statistics were computed with the non-parametric Wilcoxon rank test with continuity correction.

Supplementary Table 1: Complete list of strains used in this study (Strain name and genotype).

**Strain name**	**Genotype**
N2	Wild type (Bristol)
RB1801	*csb-1(ok2335)* X.
CB4037	*glp-1(e2141)* III.
BJS259	*glp-1(e2141)* III.;*csb-1(ok2335)* X.
SJ4103	*zcIs14*[myo-3::GFP(mit)]
SJ4143	*zcIs17*[ges-1::GFP(mit)]
BJS348	*zcIs14[myo-3::GFP(mit)];csb-1(ok2335)* X.
BJS332	*zcIs17[ges-1::GFP(mit)];csb-1(ok2335)* X.
CZ10175	*zdIs5*[mec-4::GFP+*lin-15(+)*] I.
BJS333	*zdIs5[mec-4::GFP+lin-15(+)]* I.;*csb-1(ok2335)* X.
BJS960	*sbjIs59*[pBS28(p_{csb-1}CSB-1::GFP)+pBS174(myo-2::tdTomato)];*csb-1(ok2335)* X.

Supplementary Table 2: *csb-1* mutants show less sensitivity to gentle touch than wild type in an age- and UV-dependent manner.

A

Comparison	p-value
Wild type Non-treated vs. Wild type Treated	0.0071 (**)
csb-1 Non-treated vs. *csb-1* Treated	0.0203 (*)
Wild type Non-treated vs. *csb-1* Non-treated	0.0120 (**)
Wild type Treated vs. *csb-1* Treated	0.0338 (*)

B

Comparison	p-value
Wild type Non-treated vs. Wild type Treated	0.1171
csb-1 Non-treated vs. *csb-1* Treated	0.0714
Wild type Non-treated vs. *csb-1* Non-treated	8.1340e-03 (**)
Wild type Treated vs. *csb-1* Treated	0.0046 (**)

(A) ALM and (B) PLM neurons were scored separately. Multiple linear regression best fit was used to compute the statistical significance in order to determine if overall the insensitivity of these conditions is different. * indicates p-value < 0.05, ** indicates p-value < 0.01, *** indicates p-value < 0.001, and **** indicates p-value < 0.0001.

Supplementary Table 3: Statistical significance of ALM and PLM neuronal beading score comparing the effects of UV treatment and time.

A

Neuron	ALM	ALM	PLM	PLM
Day	Day 1	Day 8	Day 1	Day 8
Wild type Non-treated vs. Wild type Treated	0.772	0.008 (**)	0.087	0.075
csb-1Non-treated vs. csb-1 Treated	0.067	0.075	0.305	0.048 (*)
Wild type Non-treated vs. csb-1 Non-treated	0.336	0.014 (**)	0.823	0.030 (*)
Wild type Treated vs. csb-1 Treated	0.075	0.102	0.009 (**)	0.019 (**)

B

Comparison	ALM neuron	PLM neuron
Wild Type Non-treated Day 0 vs. Day 7	0.659	4.457 e-07 (****)
csb-1Non-treated Day 0 vs. Day 7	0.018 (**)	6.498 e-10 (****)

(A) Compares the effects of UV treatment, while (B) compares the effects over time. Statistics were computed with the non-parametric Wilcoxon rank test with continuity correction. * indicates p-value < 0.05, ** indicates p-value < 0.01, *** indicates p-value < 0.001, and **** indicates p-value < 0.0001.

Supplementary Table 4: Statistical significance of mitochondrial network classifications.

Comparison	p-value
Wild type Non-treated vs. Wild type Treated	0.0169 (**)
csb-1 Non-treated vs. *csb-1* Treated	0.0004 (***)
Wild type Non-treated vs. *csb-1* Non-treated	0.0084 (**)

Fisher exact test for count data with Monte Carlo simulated p-value was used to compute the statistical significance between the different conditions assayed, in order to determine if overall the conditions show different classifications. * indicates p-value < 0.05, ** indicates p-value < 0.01, *** indicates p-value < 0.001, and **** indicates p-value < 0.0001.
